# Bone Formation Ability and Cell Viability Enhancement of MC3T3-E1 Cells by Ferrostatin-1 a Ferroptosis Inhibitor of Cancer Cells

**DOI:** 10.3390/ijms222212259

**Published:** 2021-11-12

**Authors:** Alireza Valanezhad, Tetsurou Odatsu, Shigeaki Abe, Ikuya Watanabe

**Affiliations:** 1 Department of Dental and Biomedical Materials Science, Graduate School of Biomedical Sciences, Nagasaki University, 1-7-1 Sakamoto, Nagasaki 852-8588, Japan; sabe_den@nagasaki-u.ac.jp (S.A.); ikuyaw@nagasaki-u.ac.jp (I.W.); 2 Department of Applied Prosthodontics, Institute of Biomedical Sciences, Nagasaki University, 1-7-1 Sakamoto, Nagasaki 852-8588, Japan

**Keywords:** ferrostatin-1, ferroptosis, erastin, regulated necrosis, bone formation

## Abstract

Recently, ferroptosis has gained scientists’ attention as an iron-related regulated necrosis. However, not many reports have investigated the effect of ferroptosis on bone. Therefore, with the present study, we assessed the effect of ferroptosis inhibition using ferrostatin-1 on the MC3T3-E1 pre-osteoblast cell. Cell images, cell viability, alkaline phosphatase activity test, alizarin red staining, and RUNX2 gene expression using real-time PCR were applied to investigate the effects of ferrostatin and erastin on MC3T3-E1 osteoblast cells. Erastin was used as a well-known ferroptosis inducer reagent. Erastin with different concentrations ranging from 0 to 50 µmol/L was used for inducing cell death. The 25 µmol/L erastin led to controllable partial cell death on osteoblast cells. Ferrostatin-1 with 0 to 40 µmol/L was used for cell doping and cell death inhibition effect. Ferrostatin-1 also displayed a recovery effect on the samples, which had already received the partially artificial cell death by erastin. Cell differentiation, alizarin red staining, and RUNX2 gene expression confirmed the promotion of the bone formation ability effect of ferrostatin-1 on osteoblast cells. The objective of this study was to assess ferrostatin-1’s effect on the MC3T3-E1 osteoblast cell line based on its ferroptosis inhibitory property.

## 1. Introduction

Bone problems such as hip fracture and dental implant loosening triggered by osteoporosis grow rapidly due to aging worldwide. Therefore, understanding the mechanism of cell death in biology is essential to prevent it [1]. The idea of exclusive cell death pathways has evolved recently from apoptosis or necrosis to several other types of regulated necrosis [2]. Apoptosis is a general mechanism of controlled cell deletion, regulating cell population [3,4]. Necrosis involves cellular dissolution following the collapse of internal homeostasis [5,6]. Cell death prevention is known as an inhibitor of some diseases [7]. Recently, a number of regulated necrosis cases with individual pathways and functions have been introduced [8]. Ferroptosis is new regulated necrosis known as iron-dependent cell death. According to Dixon, S. et al. (2013), in this type of cell death, lipid peroxidation plays a key role in cell membrane damage; instead of apoptosis and necrosis, the triggering of caspase and adenosine triphosphate was not noticed in ferroptosis [9]. In ferroptosis, the inhibition of glutathione peroxidase 4 (GPX4) and cystine/glutamate antiporter (system X_C_-) interrupts the cysteine metabolism and boosts lipid peroxidation. Excess iron through the Fenton reaction causes ferroptosis, making hydroxyl radicals from superoxide or hydrogen peroxide [10].

Iron is a necessary nutrient; however, anemia, cancer, liver, and heart diseases are caused by iron imbalance in the body [11]. Both types of iron disorders, deficiency and overload, could cause different diseases [12]. Wang, H. et al. (2017) found that iron overload could induce ferroptosis [13]. In addition, Jeney, V. et al. (2017) reported that iron overload could cause osteoporosis [14]. Therefore, controlling ferroptosis could effectively prevent osteoporosis or promote the bone formation ability of osteoblast cells. Cell death by ferroptosis is due to its reactivity as a radical trapping antioxidant [15]. Erastin is a well-known molecule that could induce ferroptosis regulated necrosis [16,17]. Cell death induced by erastin on the MC3T3-E1 preosteoblast cells was evaluated [18]. Ferrostatin-1 is also one of the effective molecules introduced as a ferroptosis cell death inhibitor [9]. Therefore, understanding the bone remodeling mechanism is necessary for calcium homeostasis, which is carried out by the collaborative engagements of osteoclasts and osteoblasts. Osteoclasts are unique cells with a bone degradation ability; their activity is regulated by different parameters such as microphthalmia transcription factor and receptor activator of nuclear factor κB ligand (RANKL) [19]. It has already been discovered that ferroptosis is related to bone diseases [20], and iron metabolism is directly engaged in bone formation or loss. For example, iron overload, an initiator of ferroptosis, could increase bone resorption and decrease bone formation [14,21]. Moreover, the iron overload could increase the number of osteoclasts [22]. Therefore, any chemical promoting bone formation or reducing the bone loss from ferroptosis could attract scientists’ attention.

Ferroptosis and bone formation pathways have one thing in common: the calcium ion (Ca^2+^). However, the role of Ca^2+^ in ferroptosis has not been well investigated. Ca^2+^ moves into mitochondria via a membrane or through an unknown transporter under oxidative stress as a permeability transition pore (PTP) opening trigger [23]. Ferroptosis pathway culminates in Ca^2+^ influx and cell death, with Ca^2+^ channels facilitating it. The release of Ca^2+^ occurs in mitochondria and the endoplasmic reticulum by activated 12-lipoxygenase and 15-lipoxygenase, passing into the cytosol from the extracellular area through Ca^2+^ channels. Cobalt chloride could act as a Ca^2+^ influx inhibitors [24] and LY83583 [25,26]. The bone mineral formation mechanism has mutual stages for ferroptosis, as mentioned above. The suggested model indicates that the ionic calcium stored in mitochondria is transported to the extracellular matrix (ECM) via vesicles before converting to the apatite with more crystallinity and propagating from dense foci [27]. Cbfa1, which belongs to the runt domain gene family, plays an essential role in osteogenesis [28]. RUNX2: RUNX family transcription factor 2 (also known as PEBP2aA or Cbfa1) is a global regulator of osteogenesis [29]. Osteoblast markers including alkaline phosphatase (ALP) and RUNX2 of Townes transgenic sickle mice show low mRNA expressions, assuming that iron overload could reduce the differentiation of osteoblasts [30]. Meanwhile, RUNX2 levels decreased in hepcidin knockdown zebrafish [31]. The studies mentioned above concluded that the osteoporotic phenotype is due to the diminished osteoblast activity and suspended mineralization. Iron downregulated the RUNX2 expression in mature human osteoblast cells [32]. This effect of iron was noted along with the decreased mineralization of the extracellular matrix of osteoblasts [14,32].

In the present study, ferrostatin-1 as a ferroptosis inhibitor reagent in cancer cells was used; then, this reagent’s enhancement of bone formation ability was investigated. Thus, the objective of the present study was to assess the effect of ferrostatin-1 on MC3T3-E1 osteoblast cells death induced by erastin (ferroptosis), viability, and differentiation and RUNX2 gene expression to evaluate the enhancement ability of the bone formation by ferrostatin-1.

## 2. Results

### 2.1. Erastin and Cell Observation

**Figure 1:**
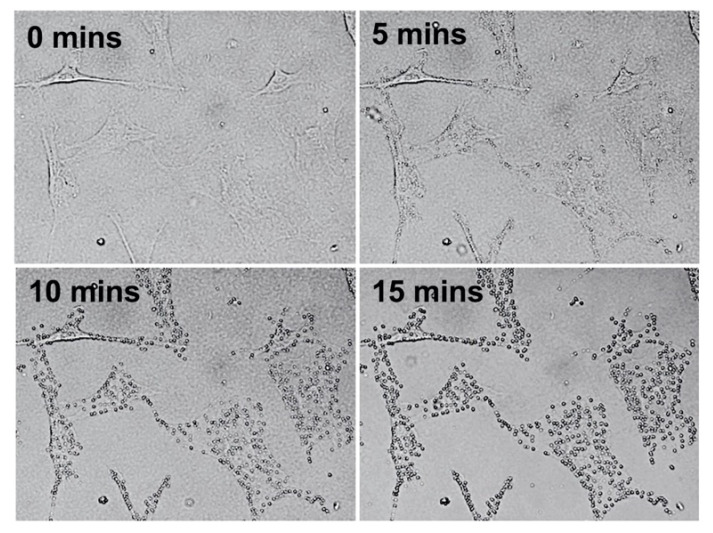
Optical microscope images of the effect of Era-50 on MC3T3-E1 cells after 0, 5, 10, and 15 min.

Figure 1 shows the optical microscope images of the effect of 50 µmol/L of erastin (Era-50) on MC3T3-E1 cells in the short period of 0, 5, 10, and 15 min. The first stage of blebbing resulting from ferroptosis (lipid peroxidation) was apparent in the cells membrane.

**Figure 2:**
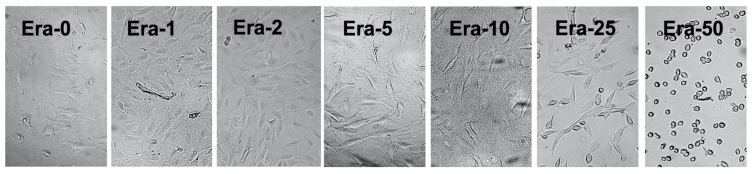
Optical microscope images of the effect of Era-0, 1, 2, 5, 10, 25, and 50 on the MC3T3-E1 cells after the 2 h incubation.

Figure 2 exhibits the optical microscope images of the MC3T3-E1 cells after the 48 h culture and incubation in the plain medium followed by the addition of 0, 1, 2, 5, 10, 25, and 50 µmol/L of erastin (Era-0, Era-1, Era-2, Era-5, Era-10, Era-25, and Era-50, respectively) for 2 h. There was no major difference among Era-0, Era-1, Era-2, Era-5, and Era-10 samples. The cells showed small dissociate adherent cells from the culture dish after 2 h for Era-25 samples. The huge detachment of adherent cells from the culture dish was observed for Era-50 after 2 h.

### 2.2. Erastin and Cell Viability

**Figure 3:**
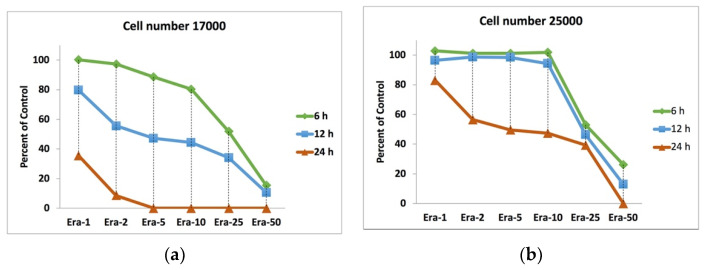
MC3T3-E1 cells viability for low and high cell numbers exposed to Era-0, Era-1, Era-2, Era-5, Era-10, Era-25, and Era-50 for 2-, 4-, and 6-h: (**a**) The results from low number (17,000 cells per well) and (**b**) the results from high number (25,000 cells per well).

Figure 3 displays the effect of the initial cell number, erastin concentration, and duration of exposure to erastin on cell proliferation or death. The MC3T3-E1 cells with the number of 17,000 (Figure 3a; low control cell number) and 25,000 (Figure 3b; high control cell number) cells/well (96-well plate) were exposed to the plain medium containing Era-0, Era-1, Era-2, Era-5, Era-10, Era-25, and Era-50 for 2, 4, and 6 h. The viability results confirmed that the effect of erastin on cell proliferation depended on the cell number. Cell proliferation started to decrease even after exposure to Era-02 after 6 h in the low control cell number sample. However, erastin had no effect on cell death for the high control cell number sample, even until exposure to Era-10 for 6 h. In all samples exposed to Era-25 for 6, 12, and 24 h, the cell death occurred partially, which was around 50 percent of the control. The cells died entirely for the low control cell number sample only by exposure to Era-05 after 24 h; meanwhile, the cells in the high cell number sample were alive even after exposure to Era-25 after 24 h. Cell death did not completely occur after the 6 and 12 h exposure to any erastin concentration below 50 µmol/L. Era-50 was enough for both low and high control cell number samples to show 100% cell death after 24 h.

### 2.3. Cell Doping and Ferrostatin-1

**Figure 4:**
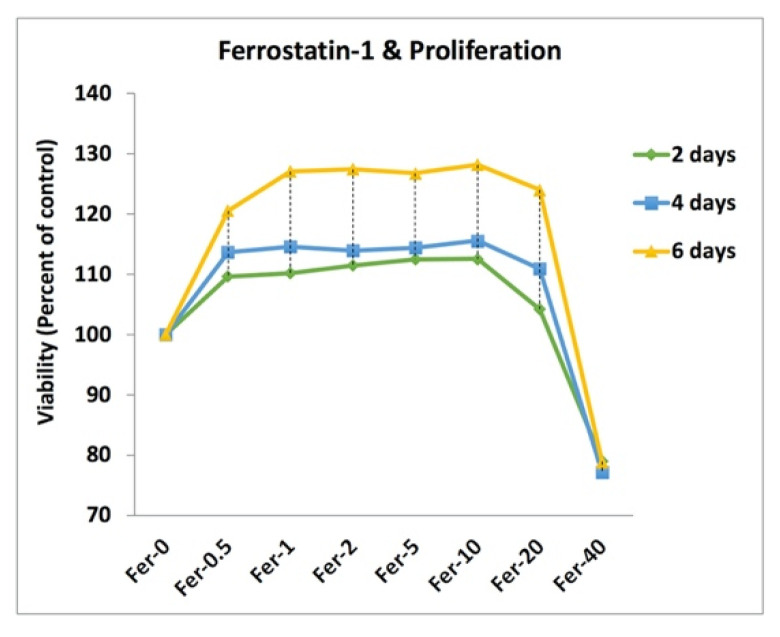
Doping effect of Fer-0, Fer-0.5, Fer-01, Fer-02, Fer-05, Fer-10, Fer-20, and Fer-40 on the MC3T3-E1 cells viability after 2, 4, and 6 days.

Figure 4 represents the effect of ferrostatin-1 on the viability of MC3T3-E1 cells after 2, 4, and 6 days of incubation. The viability results confirmed that the addition of 0, 0.5, 1, 2, 5, 10, 20, and 40 µmol/L ferrostatin-1 (Fer-0, Fer-0.5, Fer-01, Fer-02, Fer-05, Fer-10, Fer-20, and Fer-40, respectively) enhanced the viability of MC3T3-E1 cells after 2, 4, and 6 days culture. After the 6-day incubation for Fer-0.5, Fer-01, Fer-02, Fer-05, Fer-10, and Fer-20 samples, the measured cell viability showed over 120% doping on proliferation. However, the 40 µmol/L ferrostatin-1, unlike other samples with a positive effect on viability, showed a negative one on the cell proliferation for 2, 4, and 6 days. In other words, ferrostatin-1 over 20 µmol/L could induce cell death.

### 2.4. Ferrostatin-1 and Ferroptosis Inhibition

**Figure 5:**
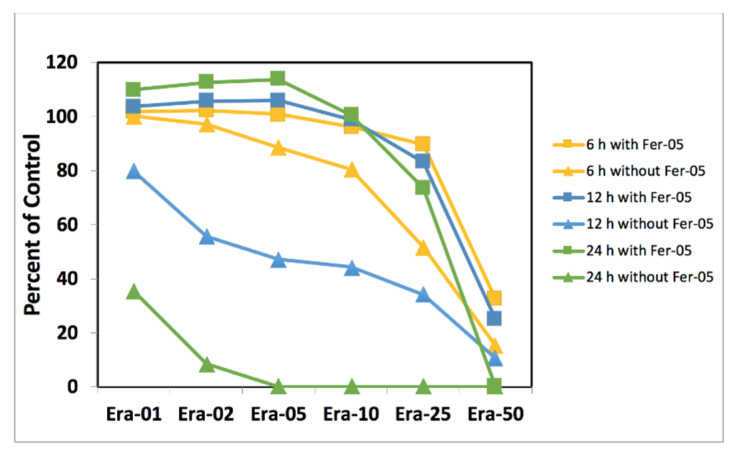
Cell death inhibition by without and with Fer-05 for Era-01, Era-02, Era-05, Era-10, Era-25, and Era-50 samples after 6-, 12-, and 24-h.

Figure 5 shows the MC3T3-E1 cell viability results for the cell death induced by low cell number samples without (Figure 5) and with (Figure 5) Ferristatin-1 addition. Fer-05 was added to Era-0, Era-1, Era-2, Era-5, Era-10, Era-25, and Era-50 containing medium for 6, 12, and 24 h. The results confirmed that the cell death by Erastin was inhibited completely after the addition of Fer-05 for Era-1, Era-2, Era-5, and Era-10 samples. According to the graph, the addition of Fer-05 could almost stop cell death for Era-25 and the cell could proliferate regardless of the cell death ability of Erastin. The critical condition in this experiment was the 100% cell death induced by 25 µmol/L Erastin. Fer-05 could eliminate the effect of Era-25 even after 24 h incubation. The cells proliferated dramatically after 24 h. The cell death was inhibited partially for Era-50 after 6 and 12 h by Fer-05. Era-50 was incubated for 24 h, but it was impossible to prevent its 100% cell death.

### 2.5. Ferrostatin-1 and Ferroptosis Recovery

**Figure 6:**
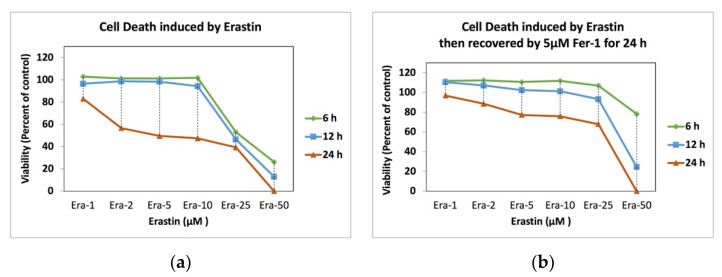
Cell death recovery before and after Fer-05 containing medium replacement: (**a**) induced by Era-0, Era-01, Era-02, Era-05, Era-10, Era-25, and Era-50 and (**b**) recovered by Fer-05 after the erastin-induced cell death.

Figure 6 demonstrates the recovery effect of ferrostatin-1 on the artificially induced cell death samples by Erastin. The cell death was induced in high cell number samples by Era-0, Era-01, Era-02, Era-05, Era-10, Era-25, and Era-50 for 6, 12, and 24 h; this was followed by removing the erastin-containing medium and adding Fer-05 medium; it was kept for a further 24-h period to be incubated. The cells were proliferated and even doped when Era-0, Era-01, Era-02, Era-05, and Era-10 medium were changed to Fer-05 for 24 h (Figure 6a). Era-25, incubated for 6, 12, and 24 h samples, was recovered after 24 h exposure to Fer-05. The 6 h incubation with the Era-50 sample also showed huge recovery after being replaced with the Fer-05 containing medium (Figure 6b).

### 2.6. ALP Activity

**Figure 7:**
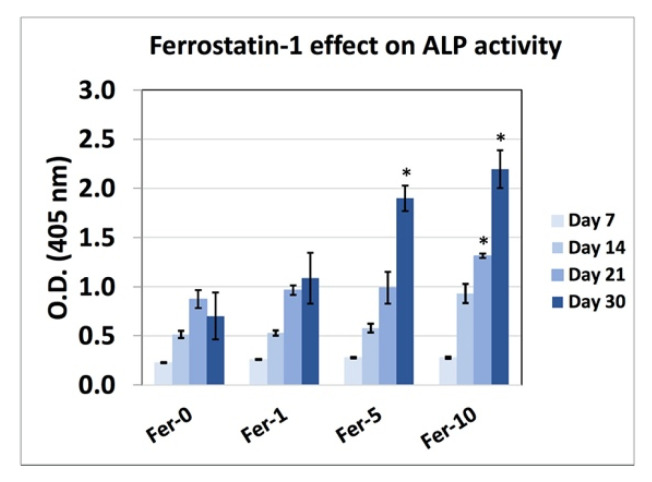
ALP activity for MC3T3-E1 cells upon exposure to Fer-0, Fer-01, Fer-05, and Fer-10 after 7, 14, 21, and 30 days of incubation. (* *p* < 0.01).

Figure 7 reveals the ALP activity results for high cell number MC3T3-E1 cells upon exposure to Fer-0, Fer-01, Fer-05, and Fer-10 after 7, 14, 21, and 30 days of incubation. Fer-05 and Fer-10 showed a higher measured ALP activity after 30 days of incubation. However, the ALP activity for Fer-10 did not show any significantly higher difference in comparison to the Fer-05 sample.

### 2.7. Alizarin Red Staining

**Figure 8:**
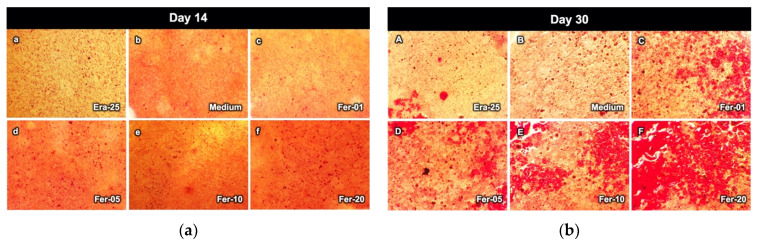
The bone nodule formation of MC3T3-E1 cells after exposure to Era-25, medium, Fer-01, Fer-05, Fer-10, and Fer-20: (**a**) after the 14 days differentiation and (**b**) after the 30 days differentiation.

Figure 8 exhibits the bone nodule formation of a high cell number sample after exposure to the Era-25 medium, Fer-01, Fer-05, Fer-10, and Fer-20, after 14 and 30 days (Figure 8b) differentiation. All samples showed small dots of calcium nodules after the 14 days incubation (Figure 8a). The bone nodules were increased in size for all samples after 30 days (Figure 8b). All ferrostatin-1 added samples showed larger calcium nodules as compared to other samples. Interestingly, erastin also showed bone nodule formation.

### 2.8. Gene Expression

**Figure 9:**
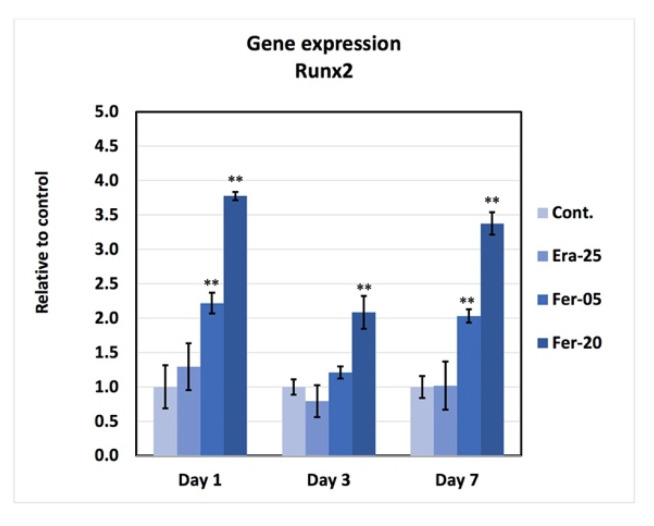
The gene expression of RUNX2 after exposure to Era-25, Fer-0, Fer-05, and Fer-20 for 1, 3, and 7 days culture with normalized data to GAPDH. (** *p* < 0.001).

Figure 9 shows the results of RT-PCR. Based on the graph, gene expressions of RUNX2 for Fer-05 and Fer-20 samples were higher than those of the control and Era-25 samples. The gene expression was significantly increased by Fer-05 on days 1 and 7, as compared to the control and Era-25 samples. The expression level of RUNX2 for Fer-20 on day 1, 3, and 7 showed a significantly higher gene expression, as compared to all other groups.

## 3. Discussion

The present study showed the inhibitory effect of ferrostatin-1 on ferroptosis, regulated cell necrosis, induced by erastin. In addition, ferrostatin-1 improved cell viability, cell differentiation, gene expression, and bone nodule formation of the MC3T3-E1 cell; the limitation of the present study is the lack of references containing the information about the bone formation effect of the calcium ion regulation in the cell death pathways such as ferroptosis.

Cell death induced by erastin is the standard method to mimic ferroptosis as regular necrosis [9,33]. This study confirmed that the effect of erastin depended on the number of cells. For example, as shown in Figure 3, all cells in the low cell number samples died 24 h after the addition of only 5 µmol/L of erastin. Meanwhile, the cells in the high cell number sample did not die entirely even after adding 25 µmol/L of erastin. The conclusion to be drawn from this phenomenon is that the calculation of erastin concentration per cell could be an accurate technique compared to the well’s erastin concentration.

The cell doping effect of ferrostatin-1 was studied in this study. The viability results showed that ferrostatin-1 could have a doping effect, even after the addition of 0.5 µmol/L of ferrostatin-1. The destructive effect of ferrostatin-1 was found when the concentration was more than 20 µmol/L. Therefore, the inhibition of ferroptosis by ferrostatin-1 depends on the concentration of that reagent; otherwise, it could be toxic in higher concentrations.

Although the ferroptosis inhibitory effect of ferrostatin-1 has already been reported [9], in this study, the effect of 5 µmol/L of ferrostatin-1 was also investigated for Era-01 to Era-50 samples. Fer-05 was selected because it was between 0.5 and 10 µmol/L concentration, which was the window with the positive effect on ferroptosis. So, Fer-05 could be effective for ferroptosis inhibition, even for the Era-25 sample.

The effect of ferrostatin-1 on the cell death inhibition of different kinds of cells such as MG63 osteoblast-like cells and Saos-2 human osteoblast-like cells has been investigate as well [34]. However, the recovery effect was not clear before this study. Therefore, the medium of the samples after artificial cell death induced by erastin was replaced by one containing different concentrations of ferrostatin. Amazingly, the viability results showed that the addition of only 5 µmol/L of erastin to the samples with a huge level of cell death could be recovered, leading to good cells proliferation. This means that ferrostatin can prevent cell death and recover the damaged cells for quicker proliferation, except for the samples containing the cells that entirely died.

The present study is the first report using the relation of calcium and ferroptosis as a bone formation effect. In other words, calcium plays the main role in the bone formation by osteoblast in the ferroptosis pathway. According to bone remodeling, any factor that could stimulate osteoclast or osteoblast could affect bone formation or osteoporosis.

Transporters for moving Ca^2+^ ions into mitochondria are still unknown and the pathway of ferroptosis culminates in the Ca^2+^ influx and Ca^2+^ channels facilitate it. The regulators of the release of Ca^2+^ ions from mitochondria and cytosol control ferroptosis and bone formation simultaneously; therefore, the bone formation ability of ferrostatin-1 was investigated in this study. The ALP activity results approved that ferrostatin-1 promoted osteoblast cells differentiation. In addition, alizarin-red images verified the ALP activity results with the bone nodule formation. Moreover, the cellular mechanism approved that RUNX2 triggers the expression of major bone matrix genes during the early stages of osteoblast differentiation [35]. RUNX2 controls the transcription of the ALP and the absence of RUNX2 results lessened bone formation, the lack of differentiated osteoblasts [28,36]. Moreover, iron is associated with RANKL/OPG ratio and osteoporosis [37], whereas iron-chelating lactoferrin has been shown to improve bone density via decreasing RANKL/OPG ratio [38]. Therefore, ferroptosis inhibitors could regulate RANKL, ALP, and RUNX2 gene expression.

## 4. Materials and Methods

### 4.1. Materials

The MC3T3-E1 osteoblast cell line (Riken Cell Bank, Tokyo, Japan) was used for cell response in this study. MEM Alpha Medium (MEM Alpha; GIBCO, InvitrogenTM, New York, NY, USA), fetal bovine serum (FBS, GIBCO), penicillin, and glutamine streptomycin (GIBCO) were used for the preparation of the medium. MTS assay (CellTiter 96 Aqueous One Solution, Promega, Madison, WI, USA) was used for the cell viability evaluation. Ascorbic acid (Sigma-Aldrich, St. Louis, MO, USA), β-glycerophosphate (Sigma-Aldrich), and dexamethasone (Sigma-Aldrich) was used for differentiation medium. Phosphate buffer solution (PBS (-) GIBCO) was used for washing the cell culture wells. ALP activity was investigated using (Takara Bio, Shiga, Japan). Erastin (Sigma-Aldrich, St. Louis, MO, USA) was used as a ferroptosis inducer. Ferrostatin-1 (Sigma-Aldrich, St. Louis, MO, USA) was applied as a ferroptosis inhibitor. The SYBR Green assay kits (SYBR Primer EX Taq II, Takara Bio) were used for real-time PCR.

### 4.2. Cell Proliferation and Observation

MC3T3-E1 cells with a density of 5000 cells/cm^2^ were seeded in the plain medium and incubated at 37 °C in saturated humidity with 95% air and 5% CO_2_. The medium was changed every two days until confluence. The cell viability was measured by adding the 20% MTS assay to the wells and the plates were incubated for further 3 h; the absorbance was measured after mixing the media and using a plate reader (Multiskan FC, Thermo Fisher, Scientific Inc., Waltham, MA, USA) at 492 nm. The cells were observed using a microscope (Nikon Eclipse TE 200, Chiyoda, Tokyo, Japan).

### 4.3. Erastin and Cell Viability

The cell viability result was investigated by adding 50 µmol/L erastin (Era-50) using the optical microscope. Later, a systematic experiment was designed to investigate the effect of the erastin concentration on the MC3T3-E1 cell death. MC3T3-E1 cells with the density of 5000 cells/cm^2^ were seeded in a 96-well plate and incubated for 24 and 48 h in saturated humidity, with 95% air and 5% CO_2_, at 37 °C. The medium was changed after 24 h (average cell number = 17,000) and 48 h (average cell number = 25,000) incubation, and the medium containing 0, 1, 2, 5, 10, 25, and 50 µmol/L erastin was added to the wells. The cells were incubated for 6, 12, and 24 h. The effect of erastin concentration and exposure time duration on the induced cell death was then investigated by this experiment.

### 4.4. Viability Doping Effect of Ferrostatin-1

MC3T3-E1 cells were seeded in the 96-well plate with a density of 5000 cells/cm^2^. Ferrostatin-1 with the concentration of 0 (control), 0.5, 1, 2, 5, 10, 20, and 40 µmol/L was added to the wells and kept in the incubator for 2, 4, and 6 days for proliferation. For the viability test, 20 percent of the MTS assay was added to each well, and the plates were incubated for further 3 h; the absorbance was measured after mixing the media and using a plate reader at 492 nm. The measured cell viability was reported in the presence of the control to investigate the doping effect of ferrostatin-1.

### 4.5. Ferrostatin-1 and Ferroptosis Inhibition

MC3T3-E1 cells with a density of 5000 cells/cm^2^ were seeded in a 96-well plate and incubated for 24 h at 37 °C in saturated humidity, with 95% air and 5% CO_2_. Era-25 was mixed with 0 and 5 µmol/L ferrostatin-1. It was added to the wells and incubated for 6, 12 and 24 h. The 20% MTS assay was added to the wells and the plates were incubated for further 3 h, and the absorbance was measured after mixing the media and using a plate reader at 492 nm. The measured cell numbers were utilized to evaluate the induced cell death level and the cell death inhibitory effect of Fer-01.

### 4.6. Cell Death Recovery

MC3T3-E1 cells were seeded with a density of 5000 cells/cm^2^ in a 96-well plate and incubated for 48 h at 37 °C in saturated humidity, with 95% air and 5% CO_2_. 25 µmol/L erastin was added to the wells and incubated for 6, 12, and 24 h to induce partial cell death. The erastin containing medium was replaced with the one having 5 µmol/L Fer-01 and incubated for 24 h to evaluate the recovery effect of ferrostatin-1. The 20% MTS assay was added to the wells and the plates were incubated for further 3 h; the absorbance was measured after mixing the media using a plate reader at 492 nm.

### 4.7. ALP Activity

MC3T3-E1 cells differentiation was evaluated using the ALP activity method. ALP activity is one of the frequently used markers of the osteoblast differentiation process. Cells were seeded in the 96-well plate with a density of 5000 cells/cm^2^ and the differentiation medium containing Fer-0, Fer-01, Fer-05, and Fer-10 were added and incubated at 37 °C, with 5% CO2, for 7, 14, 21, and 30 days. The differentiation medium was changed every 2 days. At each incubation period, the wells were washed with PBS cells denatured in 50 µL of the extracting solution; then, the buffer solution was added and kept for 15 min. The substrate solution was added and kept at 37 °C for 30 min. Finally, the stop solution was added to stop the reaction. The optical density for the evaluation of the ALP activity was obtained using a plate reader at 405 nm.

### 4.8. Alizarin Red Staining

Alizarin red staining was utilized to investigate the effect of ferrostatin-1 on the biomineralization of the MC3T3-E 1 cells after 16 and 30 days. The osteoblast cells were seeded into the wells with a density of 5000 cells/cm^2^; the differentiation medium containing Fer-0, Fer-01, Fer-05, and Fer-10 was added to the wells. After incubation, the cells were washed with PBS and fixed with 10% formaldehyde for 30 min. Wells were washed with deionized water and stained with 1% alizarin red for 15 min. Alizarin red was washed away 3 times with deionized water, and the bone nodules were observed by an optical microscope.

### 4.9. Gene Expression

MC3T3-E1 osteoblast cells were seeded in the 2 mL medium within a 6-well plate having the cell density of 5000 cells/cm^2^. The plates were incubated for 24 h; then, the medium was changed to a differentiation one containing Fer-0, Fer-01, Fer-05, Fer-10, and Fer-20; the damaged cells by ferroptosis were prepared by adding the differentiation medium containing Era-25. The plates were incubated for 1, 3, 7, and 14 days, and the medium was changed every two days. At each time point, the cells were lysed to collect mRNA (NucleoSpin RNA, Machery-Nagel, Dueren, Germany). Next, it was converted to cDNA using reverse transcriptase (GoScript Reverse Transcription System, Promega, Madison, WI, USA). The real-time PCR (RT-PCR, Thermal Cycler Dice, Takara Bio, Shiga, Japan) system was then applied for the evaluation of the gene expressions. The gene focused on this study was RUNX2. The sequence of each primer is listed in Table 1.

Data were normalized to GAPDH and expressed as the relative induction of the control at the corresponding time point.

### 4.10. Statistical Analysis

Data are presented as mean ± standard deviation. A one-way analysis of variance and *t*-test comparisons were utilized for parametric analysis, whereas the Kruskal–Wallis test was applied for nonparametric analysis. Probability values (*p*) < 0.05 were considered significant.

## 5. Conclusions

MC3T3-E1 cell death induced by ferroptosis depends on the erastin concentration per cell number ratio. Ferrostatin-1 showed an inhibitory effect on ferroptosis and MC3T3-E1 osteoblast cell death induced by erastin. Ferrostatin-1 also had a doping effect on the viability of the MC3T3-E1 cells. Induced cell death by ferroptosis could be recovered by ferrostatin-1. Ferrostatin-1, as an antiferroptosis reagent, could promote differentiation and bone nodule formation of MC3T3-E1 cells. RUNX2 gene expression confirmed the enhancement of bone-forming ability of ferrostatin-1 added MC3T3-E1 cell.

The possible future application of our knowledge concerning ferroptosis cell death inhibition may be in designing a biomaterial with superior bone formation ability compared to previous studies in this field.

## Figures and Tables

**Table 1:** Sequences of the primers used in RT-PCR.

Gene	Sequences
RUNX2	5′-CCGCACGACAACCGCACCAT-3′ and 5′-CGCTCCGGCCCACAAATCTC-3′
GAPDH	5′-ACCACAGTCCATGCCATCAC-3′ and 5′-TCCACCACCCTGTTGCTGTA-3′

## Data Availability

Not applicable.
